# Endostatin as a biomarker of systemic sclerosis: insights from a systematic review and meta-analysis

**DOI:** 10.3389/fimmu.2024.1450176

**Published:** 2024-12-23

**Authors:** Arduino A. Mangoni, Angelo Zinellu

**Affiliations:** ^1^ Discipline of Clinical Pharmacology, College of Medicine and Public Health, Flinders University, Bedford Park, SA, Australia; ^2^ Department of Clinical Pharmacology, Flinders Medical Centre, Southern Adelaide Local Health Network, Bedford Park, SA, Australia; ^3^ Department of Biomedical Sciences, University of Sassari, Sassari, Italy

**Keywords:** endostatin, systemic sclerosis, biomarkers, vascular dysfunction, ineffective angiogenesis, fibrosis, complications

## Abstract

**Introduction:**

The critical role played by vascular dysfunction and ineffective angiogenesis in the pathophysiology of systemic sclerosis (SSc) suggests that circulating biomarkers reflecting these alterations may be useful in the clinical evaluation of this patient group. We sought to address this issue by conducting a systematic review and meta-analysis of studies investigating a such candidate biomarker, endostatin, an endogenous glycoprotein exerting anti-angiogenic effects, in SSc patients and healthy controls.

**Methods:**

A literature search was conducted in the electronic databases Web of Science, PubMed, and Scopus from inception to 27 May 2024. Risk of bias and certainty of evidence were assessed using the JBI checklist for analytical studies and GRADE, respectively.

**Results:**

In 19 eligible studies, circulating endostatin concentrations were significantly higher in SSc patients than controls (standard mean difference, SMD=0.90, 95% CI 0.56 to 1.23, p<0.001; low certainty of evidence). Endostatin concentrations were also significantly higher in SSc patients with digital ulcers than those without (SMD=0.43, 95% CI 0.24 to 0.62, p<0.001; very low certainty of evidence) and in patients with pulmonary arterial hypertension than those without (SMD=1.21, 95% CI 0.67 to 1.76, p<0.001; very low certainty of evidence). By contrast, no significant differences were observed between SSc patients with limited vs. diffuse disease and those with different video capillaroscopy patterns. There was limited evidence regarding endostatin concentrations in SSc patients with interstitial lung disease, telangiectasias, and gastrointestinal manifestations. There were no significant associations in meta-regression and subgroup analysis of studies investigating endostatin in SSc patients and controls between the effect size and various patient and study characteristics.

**Discussion:**

Therefore, the results of this systematic review and meta-analysis suggest that measuring endostatin can be useful in assessing the presence of SSc and specific complications, i.e., digital ulcers and pulmonary arterial hypertension, in these patients.

**Systematic review registration:**

https://www.crd.york.ac.uk/prospero/, identifier CRD42024558174.

## Introduction

Vascular dysfunction and ineffective angiogenesis play a critical pathophysiological role in systemic sclerosis (SSc), a chronic autoimmune condition characterized by the development of skin and visceral fibrosis (1–4). Such abnormalities in vascular function and angiogenesis occur in the early stages of the disease, generally before the development of fibrosis (5, 6), and have various clinical manifestations, including the Raynaud’s phenomenon, telangiectasias, pitting scars, nailfold capillaroscopy abnormalities, digital ulcers, and pulmonary arterial hypertension (7–9). The available evidence suggests that structural and functional alterations of the endothelium caused by autoantibodies, viral agents, and oxidative stress can lead to an imbalance between vasoconstrictive and vasodilating factors (10–14). Such imbalance is associated with the increased expression of cell adhesion molecules and chemokines (15) and concomitant upregulation of pro-angiogenic, e.g., vascular endothelial growth factor (VEGF) (16), and anti-angiogenic factors, e.g., endostatin (17).

Although many studies have consistently reported upregulation and excess VEGF concentrations in SSc, studies investigating anti-angiogenic factors such as endostatin have provided conflicting results, with elevations primarily observed in SSc patients with ischemic manifestations and pulmonary arterial hypertension (18). Endostatin is a circulating glycoprotein that exerts well-known anti-angiogenic effects as a VEGF receptor blocker through its amino terminal part (19). Additionally, its carboxy-terminal part exerts significant anti-fibrotic effects (20, 21). Therefore, the combination of anti-angiogenic and anti-fibrotic effects may account, at least partially, for the inconsistent results of studies investigating this glycoprotein in SSc (18). This issue notwithstanding, recent studies using proteomic analysis have also reported that endostatin can significantly predict the clinical progression of SSc, supporting its role as a candidate biomarker in the clinical evaluation of this patient group (22).

Given the effects of endostatin on angiogenesis and fibrosis and the conflicting evidence regarding its associations with SSc, we conducted a systematic review and meta-analysis to critically appraise the available evidence regarding circulating endostatin concentrations in SSc patients and healthy controls and in SSc patients with specific disease type (limited vs. diffuse), nailfold video capillaroscopy patterns, and complications. We also investigated possible associations between the effect size of the differences in endostatin concentrations and pre-defined study and patient characteristics.

## Materials and methods

### Study selection

We searched the electronic databases Web of Science, Scopus, and PubMed from their inception to 27 May 2024 for relevant articles using the following terms: “systemic sclerosis” OR “scleroderma” OR “SSc” AND “endostatin”. Abstracts and, if relevant, full text of publications were independently assessed by two investigators (AAM and AZ). Inclusion criteria were: (i) the measurement of circulating endostatin concentrations in SSc patients diagnosed according to accepted guidelines at the time of the study conduct and healthy controls and in SSc patients with limited or diffuse disease type, specific video capillaroscopy pattern, and individual complications (i.e., digital ulcers, pulmonary arterial hypertension, interstitial lung disease, telangiectasias, renal crisis, cardiac involvement, musculoskeletal involvement, and gastrointestinal manifestations) in case-control studies (4), (ii) the inclusion of adult participants, and (iii) the availability of the full-text of the publication in the English language. The video capillaroscopy patterns were categorized as early pattern (few enlarged/giant capillaries and capillary haemorrhages, well-preserved capillary distribution, and no capillary loss), active pattern (frequent giant capillaries and capillary haemorrhages, moderate capillary loss, mildly disorganized capillary architecture, mild or absent ramified capillaries), and late pattern (irregular capillary enlargement, few or absent giant capillaries and haemorrhages, severe capillary loss and disorganization (23). Exclusion criteria were: (i) studies reporting duplicate or irrelevant information, (ii) the inclusion of participants under 18 years, and (iii) non-case-control studies. The references of each article were also hand-searched to identify additional studies.

The two investigators independently extracted the following variables from each article for further analysis: the year of publication, the details regarding the first author, the country and the continent where the study was conducted, the number of participants, the mean age, the male-to-female ratio, the mean disease duration, endostatin concentrations, the biological matrix assessed (serum or plasma), and the fraction of patients affected by diffuse or limited disease and other complications.

We assessed the risk of bias of each study using the Joanna Briggs Institute (JBI) Critical Appraisal Checklist for analytical studies (24). We evaluated the certainty of evidence for each endpoint using the GRADE Working Group system (25). We followed the Preferred Reporting Items for Systematic Reviews and Meta-Analyses (PRISMA) 2020 statement (**Supplementary Table 1**) (26), and registered the study protocol in an international register (PROSPERO registration number: CRD42024558174).

### Statistical analysis

We generated forest plots of standardized mean differences (SMDs) and 95% confidence intervals (CIs) to investigate differences in endostatin concentrations between SSc patients and healthy controls and between SSc patients with different video capillaroscopy pattern, diffuse or limited disease, and with or without complications. A p-value of less than 0.05 was considered statistically significant. If necessary, we extracted data from graphs using the Graph Data Extractor software (San Diego, CA, USA). We extrapolated the means and standard deviations from medians and interquartile ranges or full ranges using published methods (27). The heterogeneity of the SMD across studies was assessed using the Q statistic (significance level at a p-value of less than 0.10) and ranked as low (I^2^ ≤25%), moderate (25%< I^2^ <75%), or high (I^2^ ≥75%). We used a random-effects model based on the inverse-variance method in the presence of high heterogeneity (28, 29). Sensitivity analysis and publication bias were assessed using established methods (30–33).

We conducted meta-regression and subgroup analyses to investigate associations between the effect size and the following parameters: year of publication, study continent, sample size, age, male-to-female ratio, sample matrix (serum or plasma), disease form, video capillaroscopy pattern, and presence of complications. Statistical analyses were performed using Stata 14 (Stata Corp., College Station, TX, USA).

## Results

**Figure 1:**
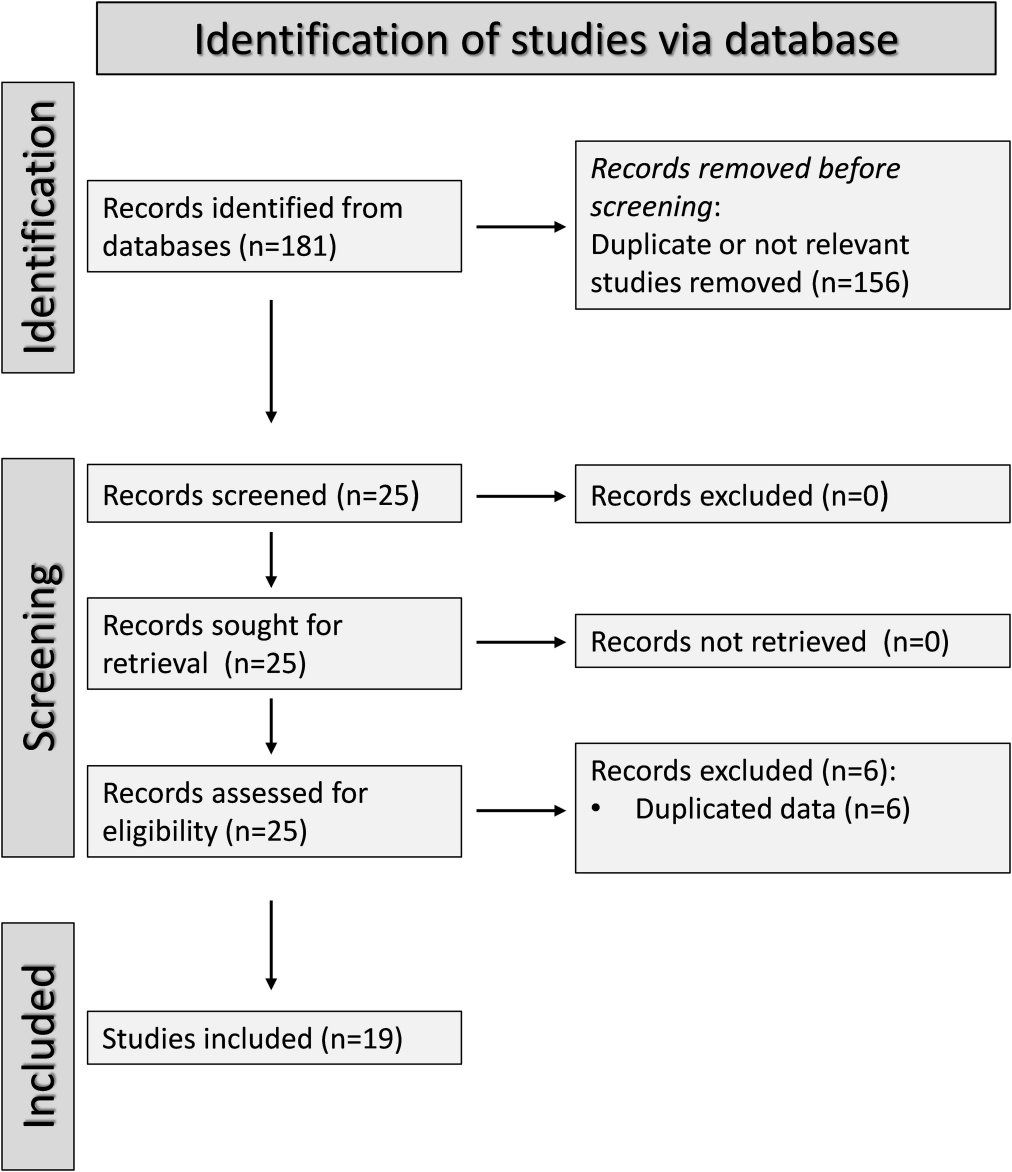
PRISMA 2020 flow diagram of study selection.

**Figure 1** shows a flow chart of the screening process and study selection. Initially, we identified 181 articles. Of them, 156 were excluded following the initial screening (duplicate data or lack of relevance for the following reasons: cellular, molecular, or animal studies, pharmacological trials or interventions outside the scope of research question, longitudinal studies without control groups, and studies without case-control or cohort design). Full-text revision of the remaining 25 articles led to the further exclusion of six (duplicate data). Therefore, 19 studies were selected for further analysis (17, 34–51) (**Table 1**). No additional studies were identified through hand-searching. There was full agreement between the two independent investigators. The risk of bias was assessed as low in 11 studies (35, 37–40, 42, 44, 46–48, 51) and moderate in the remaining eight (17, 34, 36, 41, 43, 45, 49, 50) (**Supplementary Table 2**). The initial level of the certainty of evidence was adjudicated as low because of the cross-sectional design of the selected studies.

**Table 1:** Characteristics of studies investigating endostatin in patients with systemic sclerosis and healthy controls.

		Controls				Patients with systemic sclerosis				
Study	Matrix	n	Age (Years)	M/F	Endostatin (Mean ± SD)	n	Age (Years)	M/F	Endostatin (Mean ± SD)	MDD (Years)
Hebbar M et al., 2000, France (34)	S	30	46.5	4/26	9.9 ± 9.7	50	54.05	7/43	53.2 ± 22.4	7.45
Distler O et al., 2002, Italy (35)	S	21	58.8	5/16	75 ± 65	43	56.3	8/35	197 ± 172	NR
Dziankowska-Bartkowiak B et al., 2006, Poland (36)	S	10	matched	matched	73.6 ± 25.9	34	matched	8/26	113.6 ± 60.7	NR
Hummers LK et al., 2009, USA (37)	P	27	57.5	10/17	11.7 ± 3.7	113	45.3	13/100	21.3 ± 7.8	9.6
Distler JHW et al., 2011, Switzerland (38)	S	66	42.75	22/44	17,485 ± 3,986	40	47	4/36	17,521 ± 6,121	NR
Avouac J et al., 2013, France (39)	S	20	matched	matched	188 ± 106	60	54	14/46	244 ± 138	17.25
Dunne JV et al., 2013, Canada (40)	P	40	matched	NR	136 ± 373	40	50.9	5/35	237 ± 120	7.9
Farouk HM et al., 2013, Egypt (41)	P	20	38.9	3/17	55.7 ± 20.2	25	40.3	4/21	194.9 ± 130.6	NR
Reiseter S et al., 2015, Norway (42)	S	100	NR	NR	65.1 ± 8.9	298	56	55/243	93.7 ± 27.4	4
Ribatti D et al., 2015, Italy (43)	S	8	matched	matched	12.84 ± 7.12	21	matched	4/17	17.1 ± 11	NR
Silva I et al., 2015, Portugal (44)	S	34	matched	matched	0.562 ± 0.322	77	52.95	5/72	0.671 ± 0.795	NR
Almeida I et al., 2016, Portugal (17)	S	25	NR	NR	3.68 ± 2.93	57	52	2/55	54.6 ± 38.8	NR
Delle Sedie A et al., 2017, Italy (45)	S	31	54	6/25	1.7 ± 0.94	41	53.5	1/40	4.32 ± 6.14	10.1
Gigante A et al., 2019, Italy (47)	S	10	40	0/10	66.8 ± 15	15	41	0/15	141.2 ± 63	NR

MDD, mean disease duration; M/F, male-to-female ratio; NR, not reported; P, plasma; S, serum. The unit of measure was ng/mL in all studies except for Distler JHW et al. (pg/mL) (38).

### Presence of SSc

As reported in **Table 1**, 14 studies investigated the concentrations of endostatin in 914 SSc patients (mean age 52 years, 86% females) and 442 healthy controls (mean age 49 years, 76% females) (17, 34–45, 47). Eleven studies were conducted in Europe and assessed serum (17, 34–36, 38, 39, 42–45, 47), whereas the remaining three were conducted in other continents and assessed plasma (37, 40, 41). Disease duration was reported in six studies and ranged between 4 and 17.25 years (34, 37, 39, 40, 42, 45). The risk of bias was low in eight studies (35, 37–40, 42, 44, 47) and moderate in the remaining six (17, 34, 36, 41, 43, 45) (**Supplementary Table 2**).

**Figure 2:**
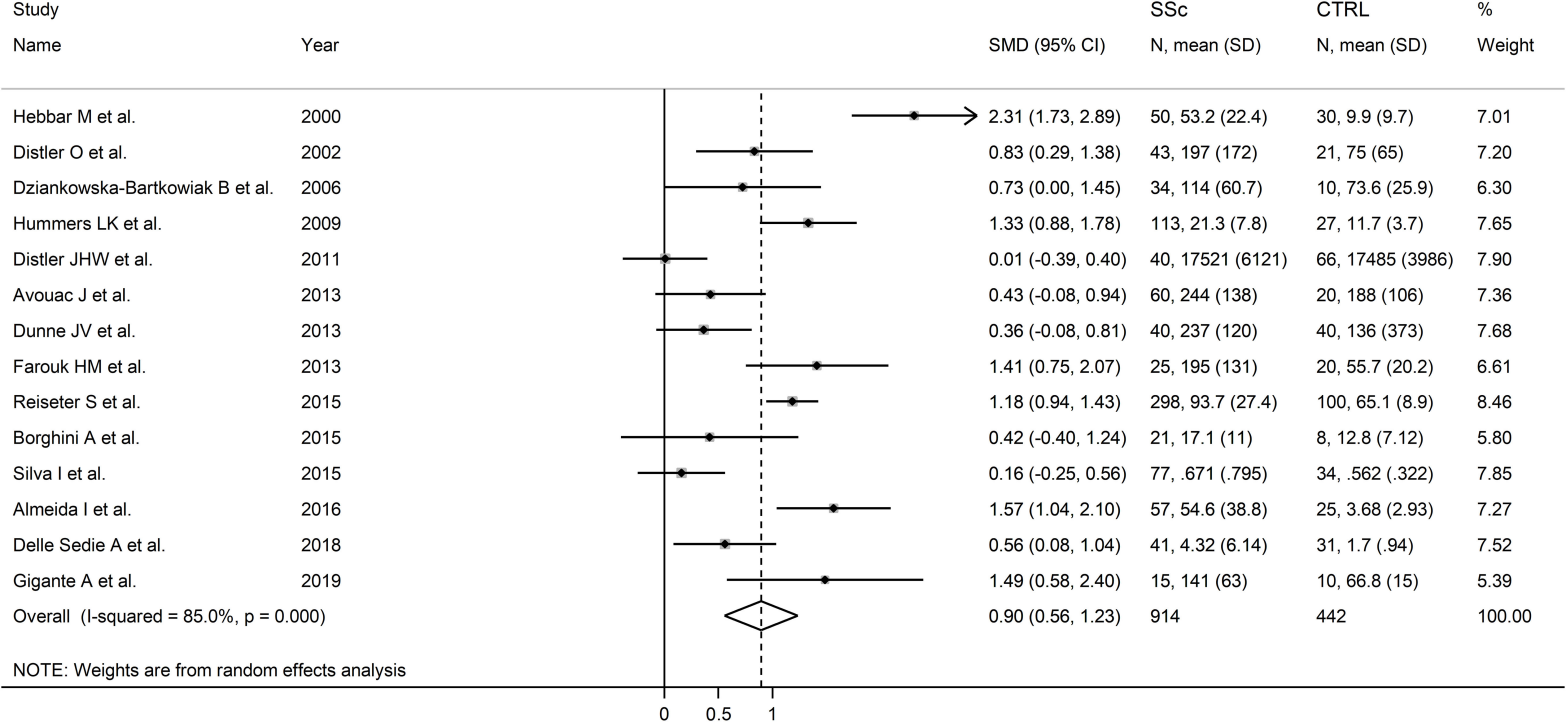
Forest plot of studies investigating endostatin concentrations in patients with systemic sclerosis and healthy controls. The forest plot displays the standardized mean differences (SMDs) and their 95% confidence intervals for each study included in the meta-analysis. Each square represents the effect size of an individual study, with the size of the square proportional to the study’s weight in the analysis. The horizontal lines indicate the 95% confidence intervals for each study, and the vertical line at 0 represents the null effect. The overall pooled effect size is represented by the diamond at the bottom of the plot, with the width of the diamond reflecting the confidence interval. Studies with confidence intervals crossing the null line (0) indicate non-significant results.

**Figure 3:**
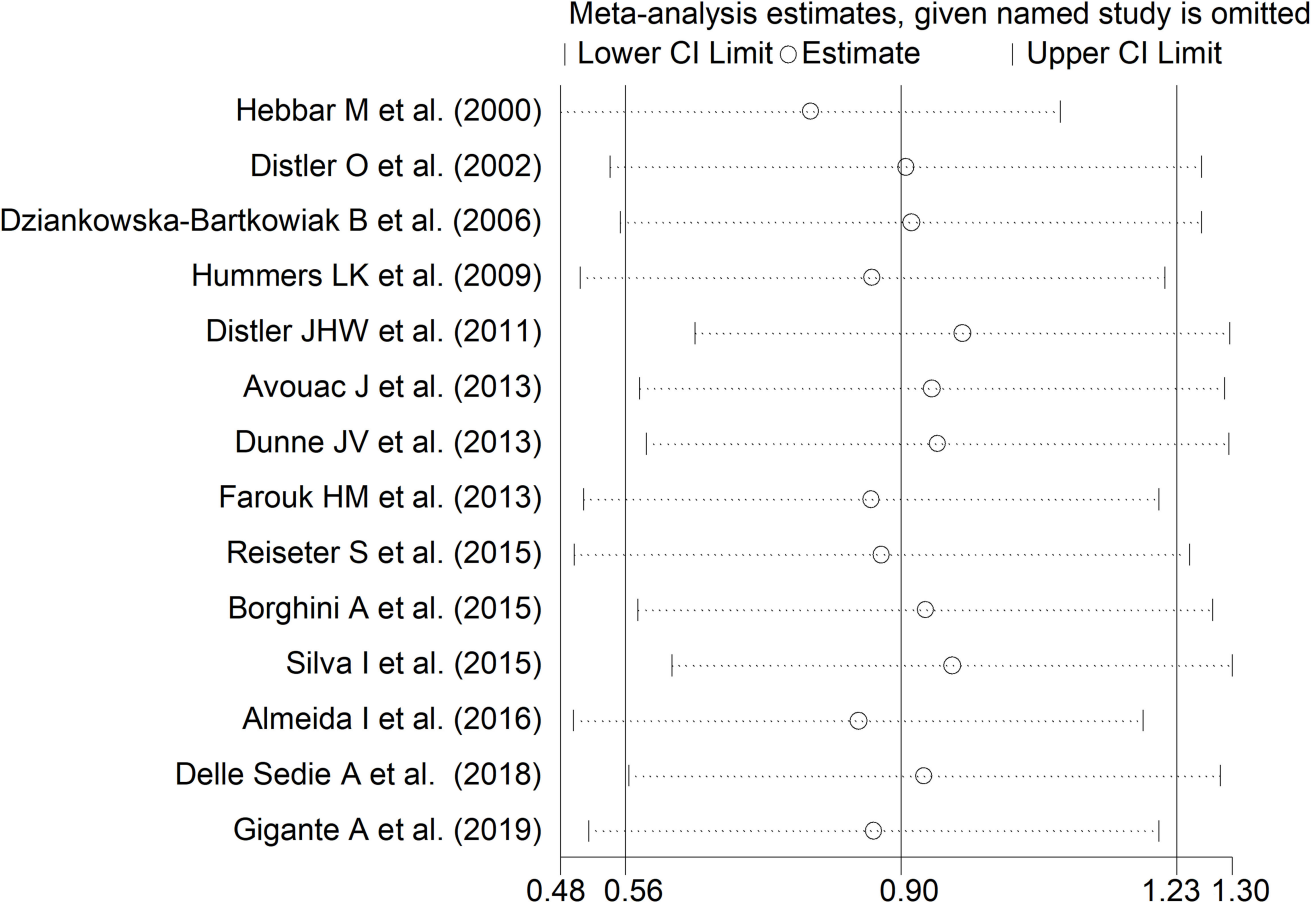
Sensitivity analysis of the association between endostatin concentrations and systemic sclerosis. For each study, the displayed effect size (hollow circles) and horizontal lines represent the overall effect size and 95% confidence intervals calculated from a meta-analysis excluding that study. The central vertical line represents the overall SMD whereas the lateral vertical lines represent the overall 95% confidence intervals.

The forest plot showed that endostatin concentrations were significantly higher in SSc patients than in controls (SMD=0.90, 95% CI 0.56 to 1.23, p<0.001; I^2^ = 85.0%, p<0.001; **Figure 2**). The pooled SMD values were stable in sensitivity analysis, ranging between 0.79 and 0.97 (**Figure 3**).

**Figure 4:**
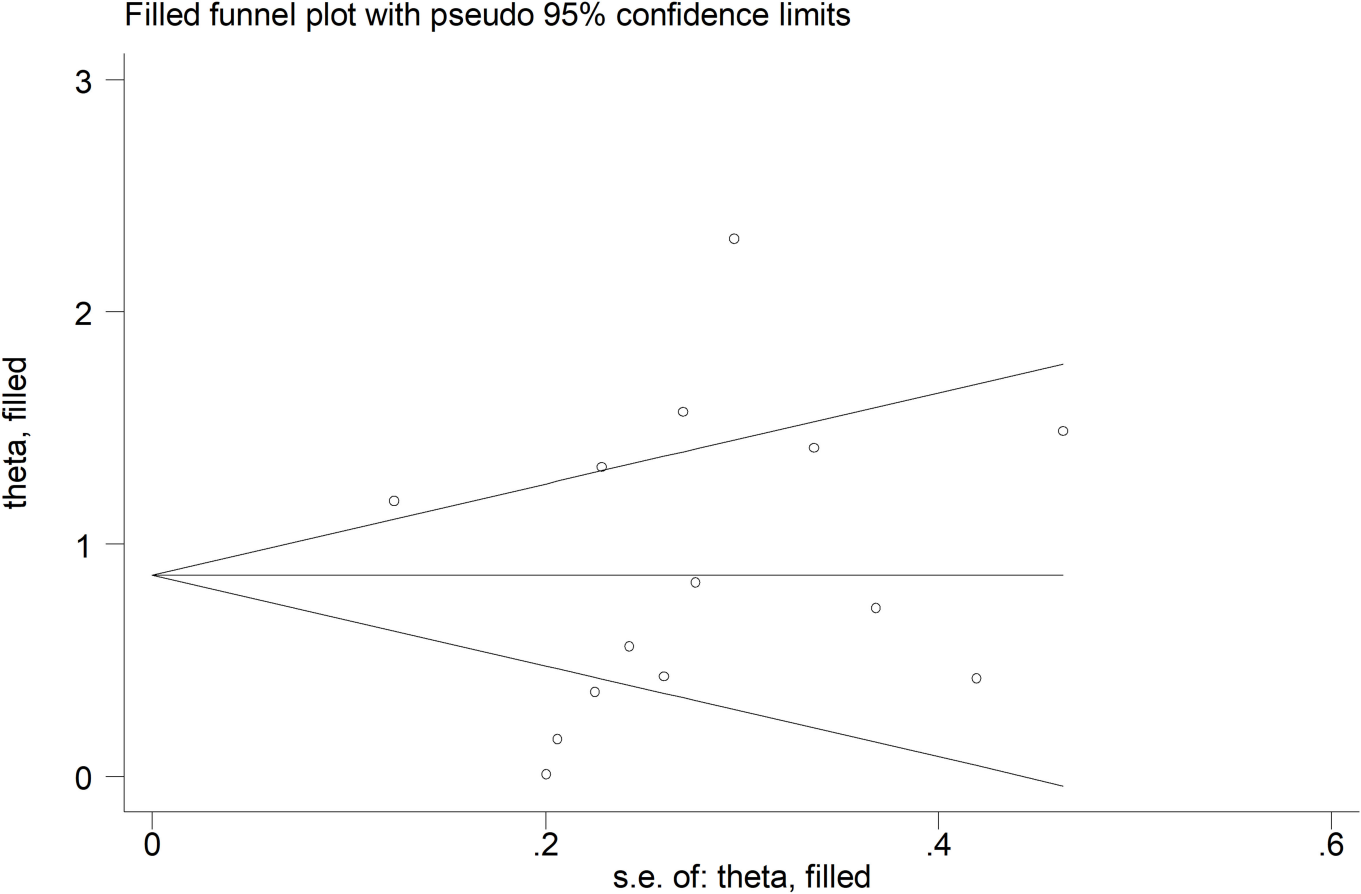
Funnel plot of studies investigating the association between endostatin concentrations and systemic sclerosis after “trimming and filling”. Dummy studies and genuine studies are represented by enclosed circles and free circles, respectively.

There was no significant publication bias (Begg’s test, p=0.27; Egger’s test, p=0.87), and no missing study was required to ensure symmetry (**Figure 4**). The resulting effect size was attenuated yet still significant (SMD=0.56, 95% CI 0.30 to 0.82, p<0.001).

There were no significant associations in meta-regression between the effect size and age (t=0.34, p=0.74), male-to-female ratio (t=0.57, p=0.58), year of publication (t=-1.15, p=0.27), or number of participants (t=0.30, p=0.77). Sub-group analysis showed no significant differences (p=0.73) in pooled SMD between studies conducted in Europe and assessing serum (SMD=0.86, 95% CI 0.46 to 1.27, p<0.001; I^2^ = 86.7%, p<0.001) and those conducted elsewhere and assessing plasma (SMD=1.02, 95% CI 0.32 to 1.72, p=0.004; I^2^ = 85.0%, p<0.001; **Supplementary Figure 1**).

The overall level of certainty remained low (level 2) after considering the low-moderate risk of bias in all studies (no change), the high and unexplained heterogeneity (downgrade one level), the lack of indirectness (no change), the large effect size (SMD=0.90, upgrade one level) (52), and the absence of publication bias (no change).

### Limited vs. diffuse disease

As reported in **Table 2**, seven studies investigated endostatin in 185 SSc patients with diffuse form and 383 with limited form (17, 35, 36, 38, 40–42). Five studies were conducted in Europe and assessed serum (17, 35, 36, 38, 42), whereas the remaining two were conducted elsewhere and assessed plasma (40, 41). The risk of bias was low in four studies (35, 38, 40, 42) and moderate in the remaining three (17, 36, 41) (**Supplementary Table 2**).

**Table 2:** Characteristics of studies investigating endostatin in patients with systemic sclerosis with limited vs. diffuse form.

Study	Limited		Diffuse		Matrix	MDD (years)
	n	Endostatin (Mean ± SD)	n	Endostatin (Mean ± SD)		
Distler O et al., 2002, Italy (35)	20	174 ± 171	23	197 ± 194	S	NR
Dziankowska-Bartkowiak B et al., 2004, Poland (36)	19	101.9 ± 53.1	15	127.1 ± 67.8	S	NR
Distler JH et al., 2011, Switzerland (38)	20	18,853 ± 6,388	20	16,188 ± 5,869	S	NR
Dunne JV et al., 2013, Canada (40)	26	237 ± 122	14	224 ± 97	P	7.9
Farouk HM et al., 2013, Egypt (41)	15	196.9 ± 150.8	10	191 ± 120.3	P	NR
Reiseter S et al., 2015, Norway (42)	220	91.1 ± 23.7	78	101.1 ± 34.1	S	4
Almeida I et al., 2016, Portugal (17)	34	28.9 ± 17.5	13	57 ± 51.6	S	NR

MDD, mean disease duration; NR, not reported; P, plasma; S, serum. The unit of measure was ng/mL in all studies except for Distler JHW et al. (pg/mL) (38).

**Figure 5:**
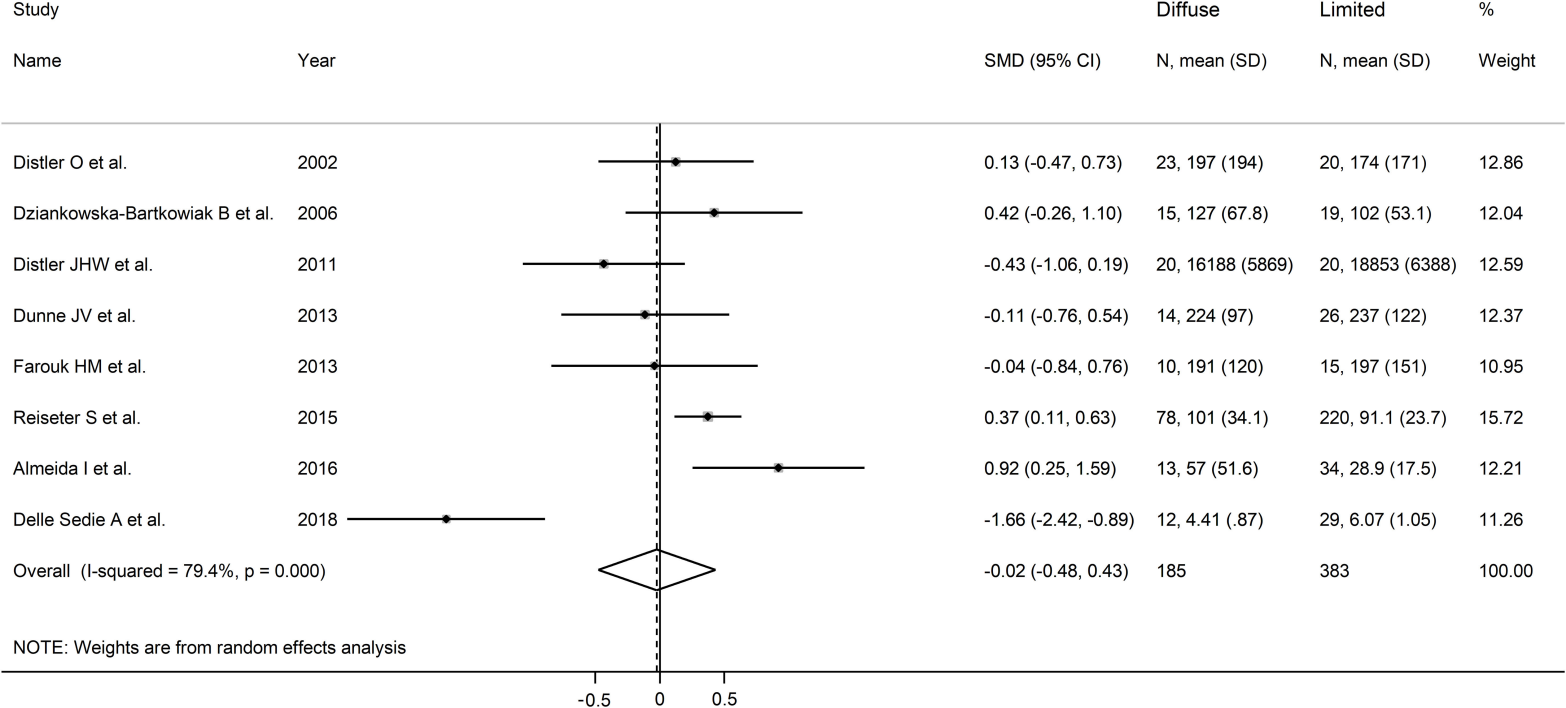
Forest plot of studies investigating endostatin concentrations in patients with systemic sclerosis with diffuse or limited form. The forest plot displays the standardized mean differences (SMDs) and their 95% confidence intervals for each study included in the meta-analysis. Each square represents the effect size of an individual study, with the size of the square proportional to the study’s weight in the analysis. The horizontal lines indicate the 95% confidence intervals for each study, and the vertical line at 0 represents the null effect. The overall pooled effect size is represented by the diamond at the bottom of the plot, with the width of the diamond reflecting the confidence interval. Studies with confidence intervals crossing the null line (0) indicate non-significant results.

**Figure 6:**
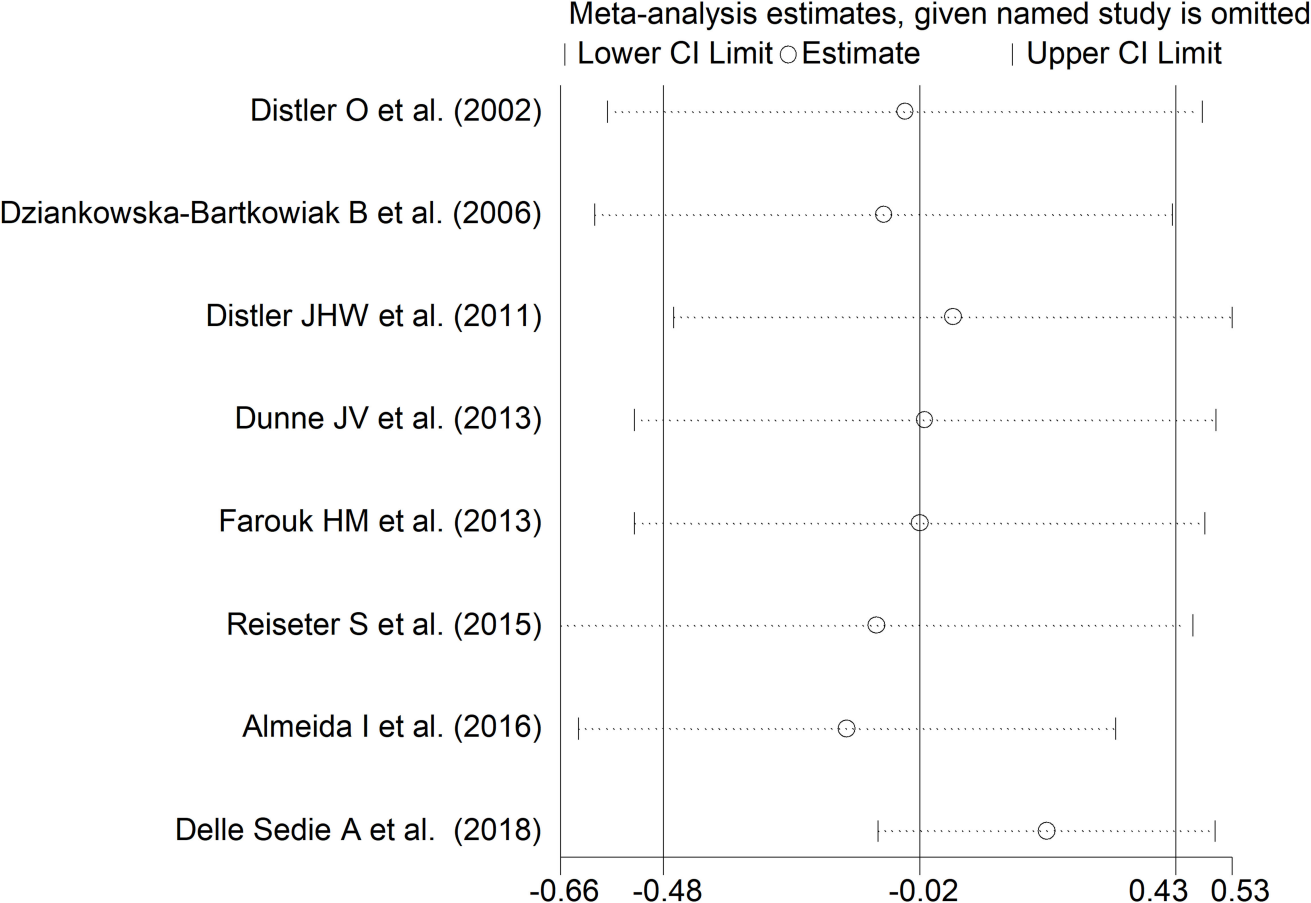
Sensitivity analysis of the association between endostatin concentration and disease form. For each study, the displayed effect size (hollow circles) and horizontal lines represent the overall effect size and 95% confidence intervals calculated from a meta-analysis excluding that study. The central vertical line represents the overall SMD whereas the lateral vertical lines represent the overall 95% confidence intervals.

The forest plot showed non-significant between-group differences in endostatin concentrations (SMD=-0.02, 95% CI -0.48 to 0.43, p=0.92, I^2^ = 79.4%, p<0.001; **Figure 5**). The results were stable in sensitivity analysis (effect size ranged between -0.15 and 0.20; **Figure 6**). The overall level of the certainty of evidence was downgraded to very low (level 1) as the assessment of publication bias, meta-regression and sub-group analysis could not be performed because of the small number of studies.

### Capillaroscopy pattern

As shown in **Table 3**, four European studies reported endostatin concentrations in SSc patients with different video capillaroscopy pattern (17, 35, 39, 46). The risk of bias was low in three studies and moderate in the remaining one (**Supplementary Table 2**).

**Table 3:** Characteristics of studies investigating endostatin in patients with systemic sclerosis according to capillaroscopy pattern.

Study	Early		Active		Late		Matrix	MDD (years)
	n	Endostatin (Mean ± SD)	n	Endostatin (Mean ± SD)	n	Endostatin (Mean ± SD)		
Distler O et al., 2002, Italy (35)	6	232 ± 290	22	130 ± 131	14	198 ± 218	S	NR
Avouac J et al., 2013, France (39)	44	234 ± 89	22	128 ± 32	24	246 ± 164	S	17.25
Almeida I et al., 2016, Portugal (17)	12	31.9 ± 20.8	21	55 ± 46.7	18	22.8 ± 10.6	S	NR
Gigante A et al., 2018, Italy (46)	22	98.9 ± 40.8	35	114.7 ± 35.5	33	133.8 ± 34.5	S	9

MDD, mean disease duration; NR, not reported; P, plasma; S, serum. The unit of measure was ng/mL in all studies.

**Figure 7:**
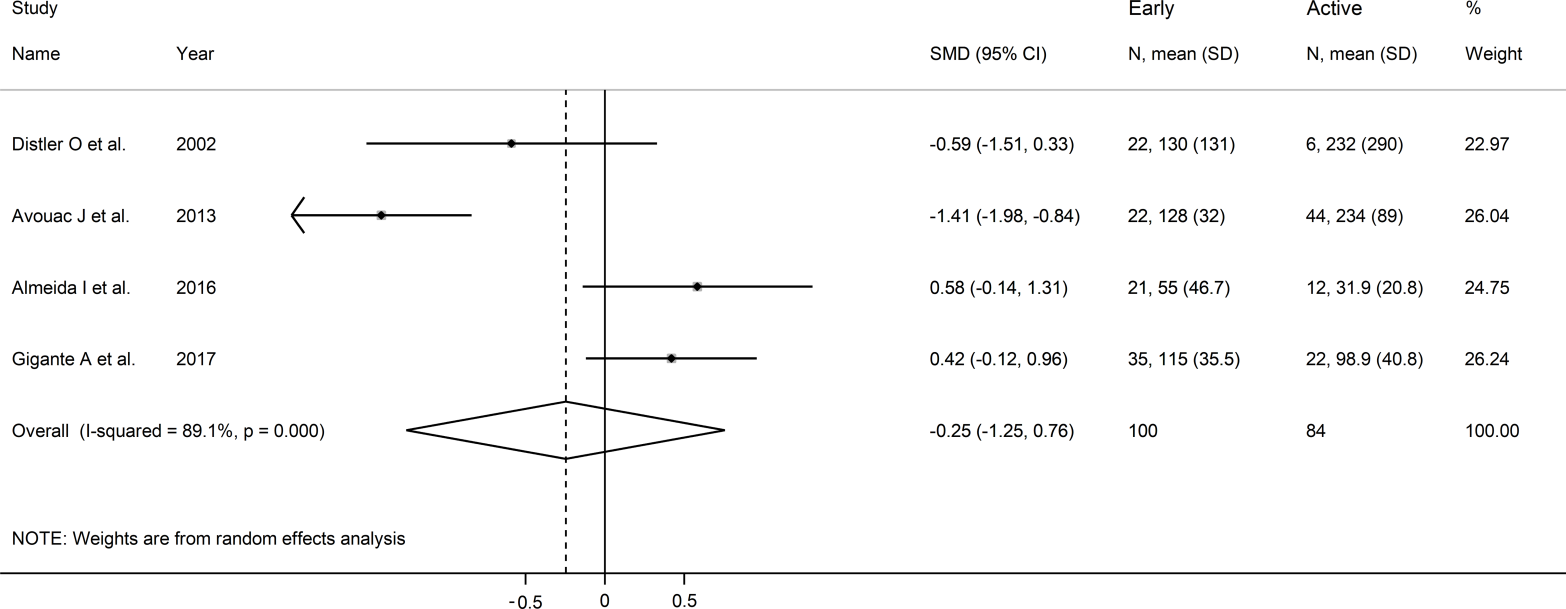
Forest plot of studies investigating endostatin concentrations in patients with systemic sclerosis with early and active capillaroscopy pattern. The forest plot displays the standardized mean differences (SMDs) and their 95% confidence intervals for each study included in the meta-analysis. Each square represents the effect size of an individual study, with the size of the square proportional to the study’s weight in the analysis. The horizontal lines indicate the 95% confidence intervals for each study, and the vertical line at 0 represents the null effect. The overall pooled effect size is represented by the diamond at the bottom of the plot, with the width of the diamond reflecting the confidence interval. Studies with confidence intervals crossing the null line (0) indicate non-significant results.

**Figure 8:**
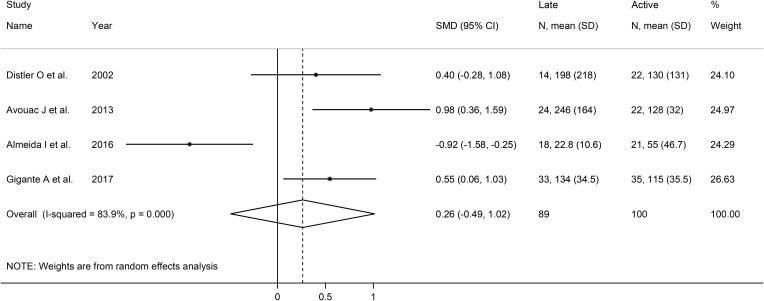
Forest plot of studies investigating endostatin concentrations in patients with systemic sclerosis with active and late capillaroscopy pattern. The forest plot displays the standardized mean differences (SMDs) and their 95% confidence intervals for each study included in the meta-analysis. Each square represents the effect size of an individual study, with the size of the square proportional to the study’s weight in the analysis. The horizontal lines indicate the 95% confidence intervals for each study, and the vertical line at 0 represents the null effect. The overall pooled effect size is represented by the diamond at the bottom of the plot, with the width of the diamond reflecting the confidence interval. Studies with confidence intervals crossing the null line (0) indicate non-significant results.

**Figure 9:**
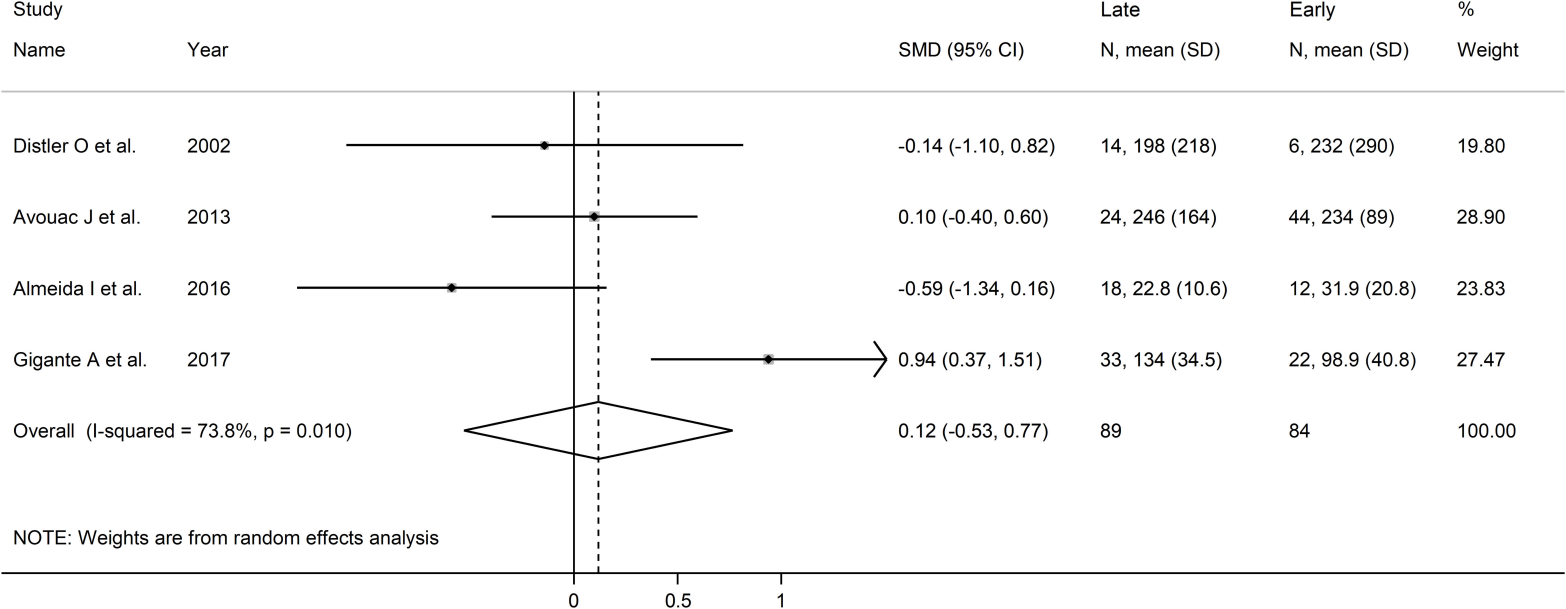
Forest plot of studies investigating endostatin concentrations in patients with systemic sclerosis with early and late capillaroscopy pattern. The forest plot displays the standardized mean differences (SMDs) and their 95% confidence intervals for each study included in the meta-analysis. Each square represents the effect size of an individual study, with the size of the square proportional to the study’s weight in the analysis. The horizontal lines indicate the 95% confidence intervals for each study, and the vertical line at 0 represents the null effect. The overall pooled effect size is represented by the diamond at the bottom of the plot, with the width of the diamond reflecting the confidence interval. Studies with confidence intervals crossing the null line (0) indicate non-significant results.

Forest plots showed no significant between-group differences in endostatin concentrations in SSc patients with early (n=84) and active (n=100) form (SMD=-0.25, 95% CI -1.25 to 0.76, p=0.63; I^2^ = 89.1%, p<0.001; **Figure 7**), active (n=100) and late (n=89) form (SMD=0.26, 95% CI -0.49 to 1.02, p=0.49; I^2^ = 83.9%, p<0.001; **Figure 8**), and late (n=89) and early (n=84) form (SMD=0.12, 95% CI -0.53 to 0.77, p=0.72; I^2^ = 70.3%, p=0.010; **Figure 9**).

Assessment of sensitivity, publication bias, meta-regression and sub-group analysis could not be performed because of the small number of studies, with a consequent downgrading of the overall level of the certainty of evidence to very low (level 1).

### Digital ulcers

As described in **Table 4**, five studies reported endostatin concentrations in 489 SSc patients, 311 without and 178 with digital ulcers (35, 42, 44, 46, 48). All studies were conducted in Europe except one, which was conducted in America (48). All studies had low risk of bias (**Supplementary Table 2**).

**Table 4:** Characteristics of studies investigating endostatin in patients with systemic sclerosis according to specific complications.

Study	Absence of complications		Presence of complications		Matrix	MDD (years)
	n	Endostatin (Mean ± SD)	n	Endostatin (Mean ± SD)		
*Digital ulcers*						
Distler O et al., 2002, Italy (35)	27	195 ± 187	16	199 ± 211	S	NR
Reiseter S et al., 2015, Norway (42)	105	79.2 ± 14.8	50	89.6 ± 19.3	S	4
Silva I et al., 2015, Portugal (44)	39	0.453 ± 0.469	38	0.895 ± 1.13	S	NR
Gigante A et al., 2018, Italy (46)	58	116.3 ± 39.7	32	127 ± 31.1	S	9
Mecoli AC et al., 2019, USA (48)	82	130.76 ± 39.00	42	145.14 ± 37.00	S	NR
*Pulmonary arterial hypertension*						
Hummers LK et al., 2009, USA (37)	91	19.5 ± 1.9	22	24.3 ± 2.3	P	9.6
Reiseter S et al., 2015, Norway (42)	263	90.6 ± 26.7	24	119.9 ± 25.2	S	4
Bauer Y et al. (a) 2021 Switzerland (50)	80	101 ± 47	77	133 ± 33	S	NR
Bauer Y et al. (b) 2021, Switzerland (50)	22	64 ± 31	22	104 ± 37	S	NR
Lemmers JMJ et al., 2023, Netherlands (51)	41	43 ± 25	40	59 ± 21	S	9.6

MDD, mean disease duration; NR, not reported; P, plasma; S, serum. The unit of measure was ng/mL in all studies.

**Figure 10:**
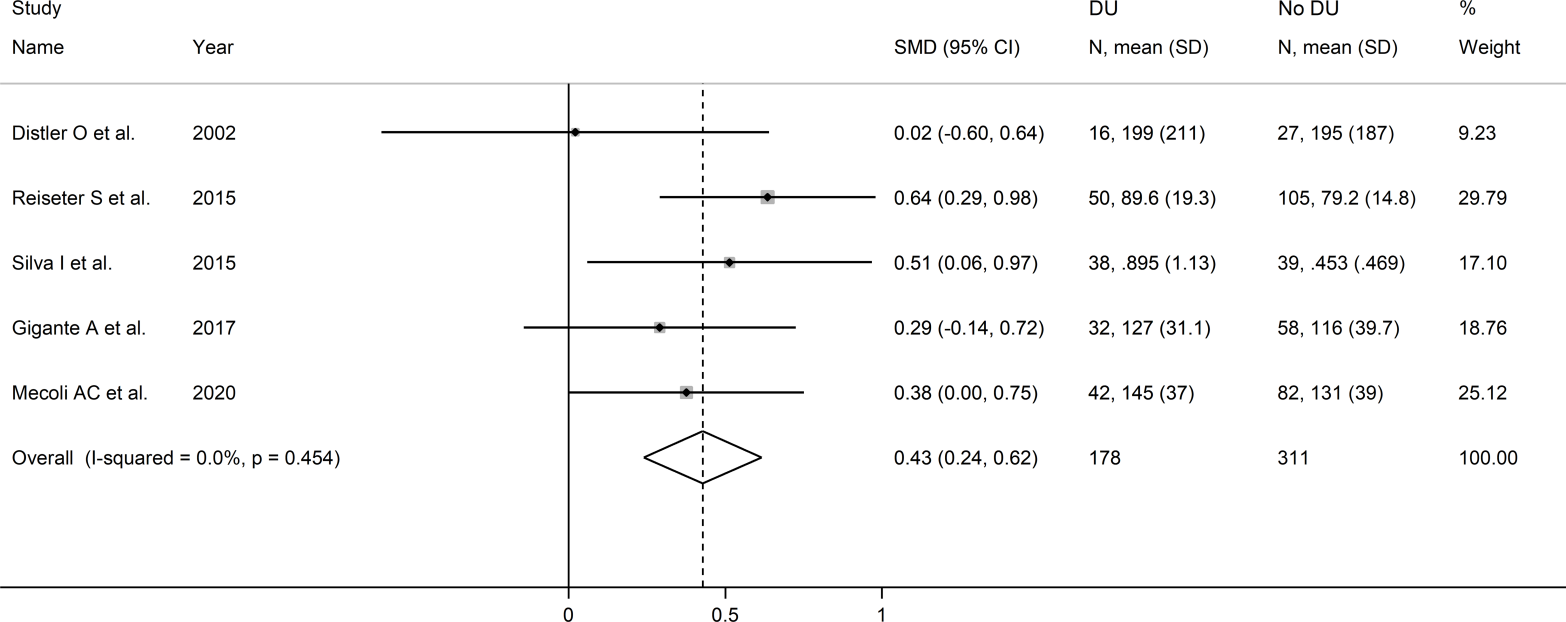
Forest plot of studies investigating endostatin concentrations in patients with systemic sclerosis with or without digital ulcers. The forest plot displays the standardized mean differences (SMDs) and their 95% confidence intervals for each study included in the meta-analysis. Each square represents the effect size of an individual study, with the size of the square proportional to the study’s weight in the analysis. The horizontal lines indicate the 95% confidence intervals for each study, and the vertical line at 0 represents the null effect. The overall pooled effect size is represented by the diamond at the bottom of the plot, with the width of the diamond reflecting the confidence interval. Studies with confidence intervals crossing the null line (0) indicate non-significant results.

**Figure 11:**
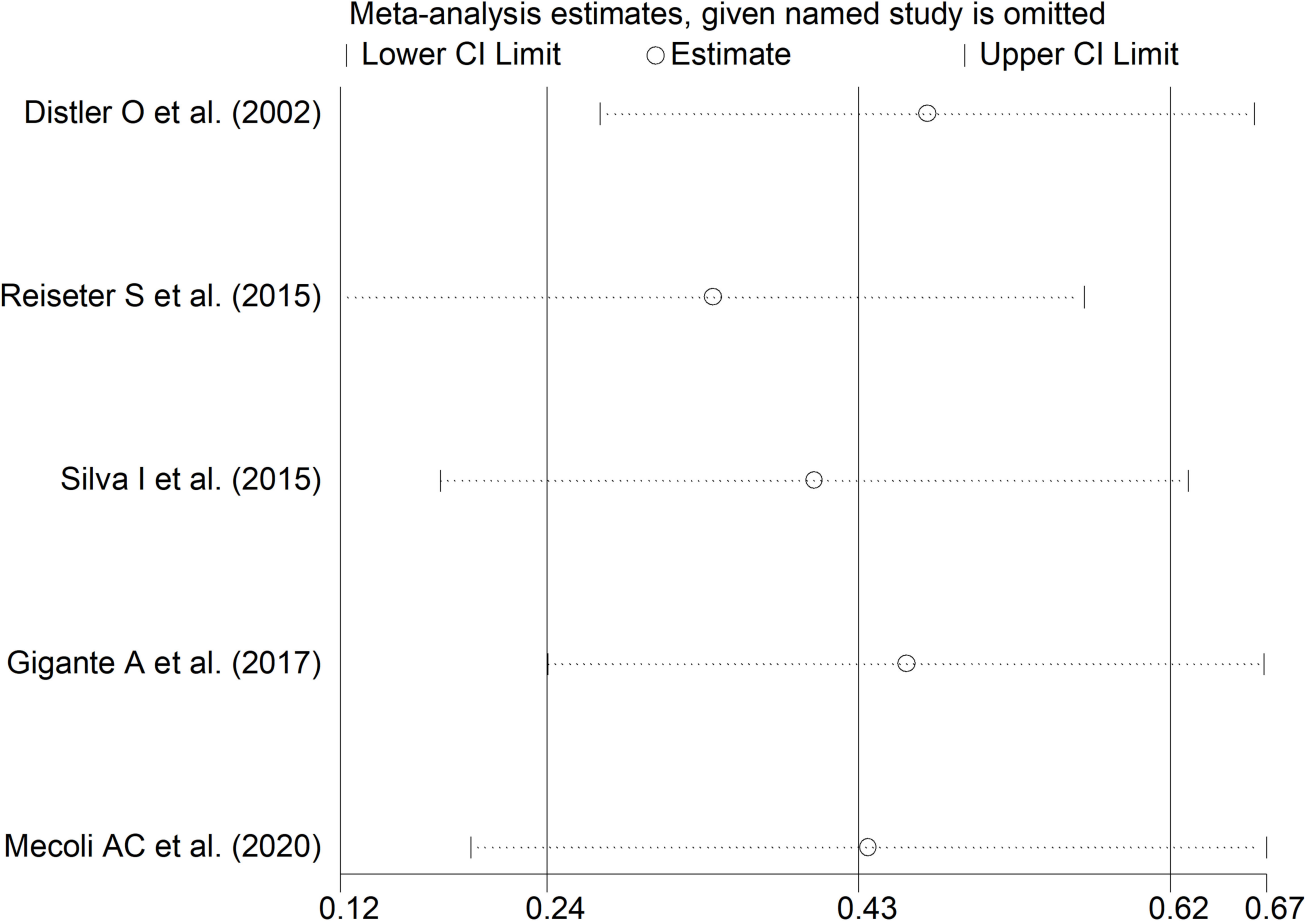
Sensitivity analysis of the association between endostatin concentrations and digital ulcers. For each study, the displayed effect size (hollow circles) and horizontal lines represent the overall effect size and 95% confidence intervals calculated from a meta-analysis excluding that study. The central vertical line represents the overall SMD whereas the lateral vertical lines represent the overall 95% confidence intervals.

The forest plot showed that SSc patients with digital ulcers had significantly higher endostatin concentrations than those without (SMD=0.43, 95% CI 0.24 to 0.62, p<0.001; I^2^ = 0.0%, p=0.004; **Figure 10**). Sensitivity analysis showed stability of the results, with an effect size ranging between 0.34 and 0.47 (**Figure 11**). Assessment of publication bias, meta-regression and sub-group analysis could not be performed because of the small number of studies, which led to a downgrading of the overall level of the certainty of evidence to very low (level 1).

### Pulmonary arterial hypertension

As shown in **Table 4**, four studies, including five group comparators, investigated endostatin concentrations in 682 SSc patients, 497 without and 185 with pulmonary arterial hypertension (37, 42, 50, 51). All studies were conducted in Europe except one, which was conducted in America (37). The risk of bias was low in three studies (37, 42, 51) and moderate in the remaining one (50) (**Supplementary Table 2**).

**Figure 12:**
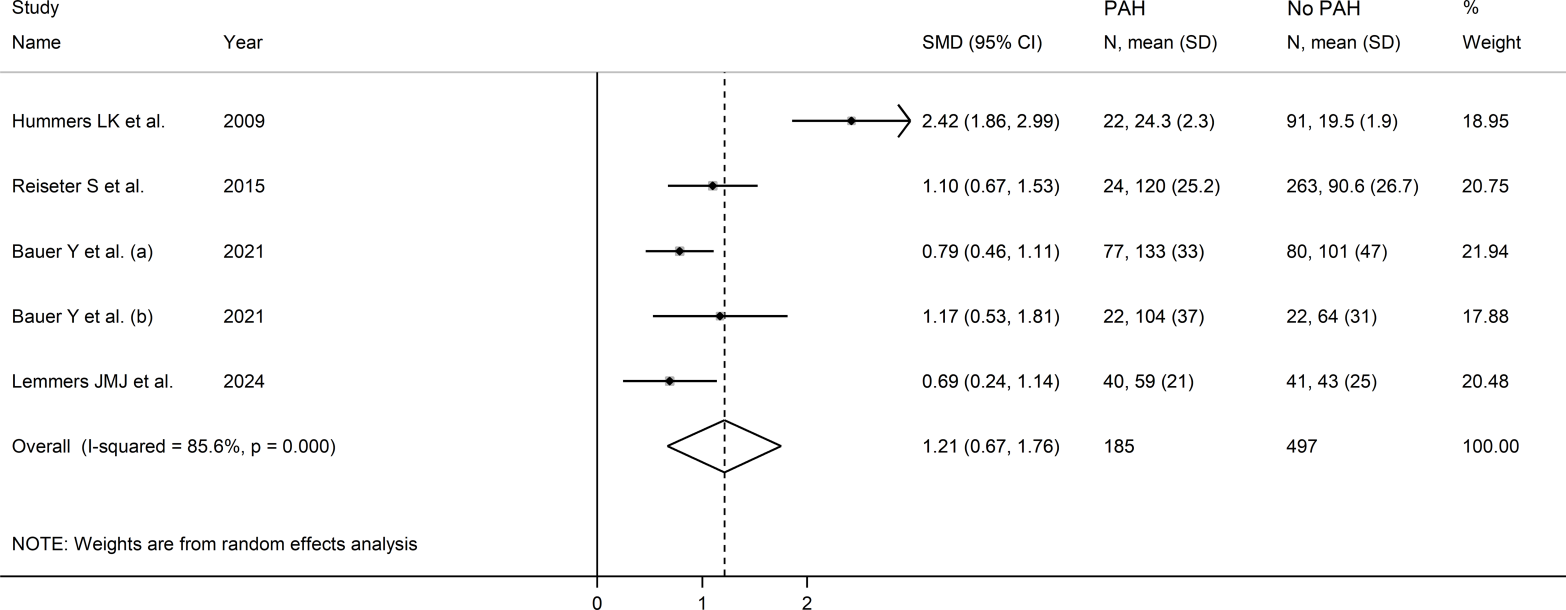
Forest plot of studies investigating endostatin concentrations in patients with systemic sclerosis with or without pulmonary arterial hypertension. The forest plot displays the standardized mean differences (SMDs) and their 95% confidence intervals for each study included in the meta-analysis. Each square represents the effect size of an individual study, with the size of the square proportional to the study’s weight in the analysis. The horizontal lines indicate the 95% confidence intervals for each study, and the vertical line at 0 represents the null effect. The overall pooled effect size is represented by the diamond at the bottom of the plot, with the width of the diamond reflecting the confidence interval. Studies with confidence intervals crossing the null line (0) indicate non-significant results.

**Figure 13:**
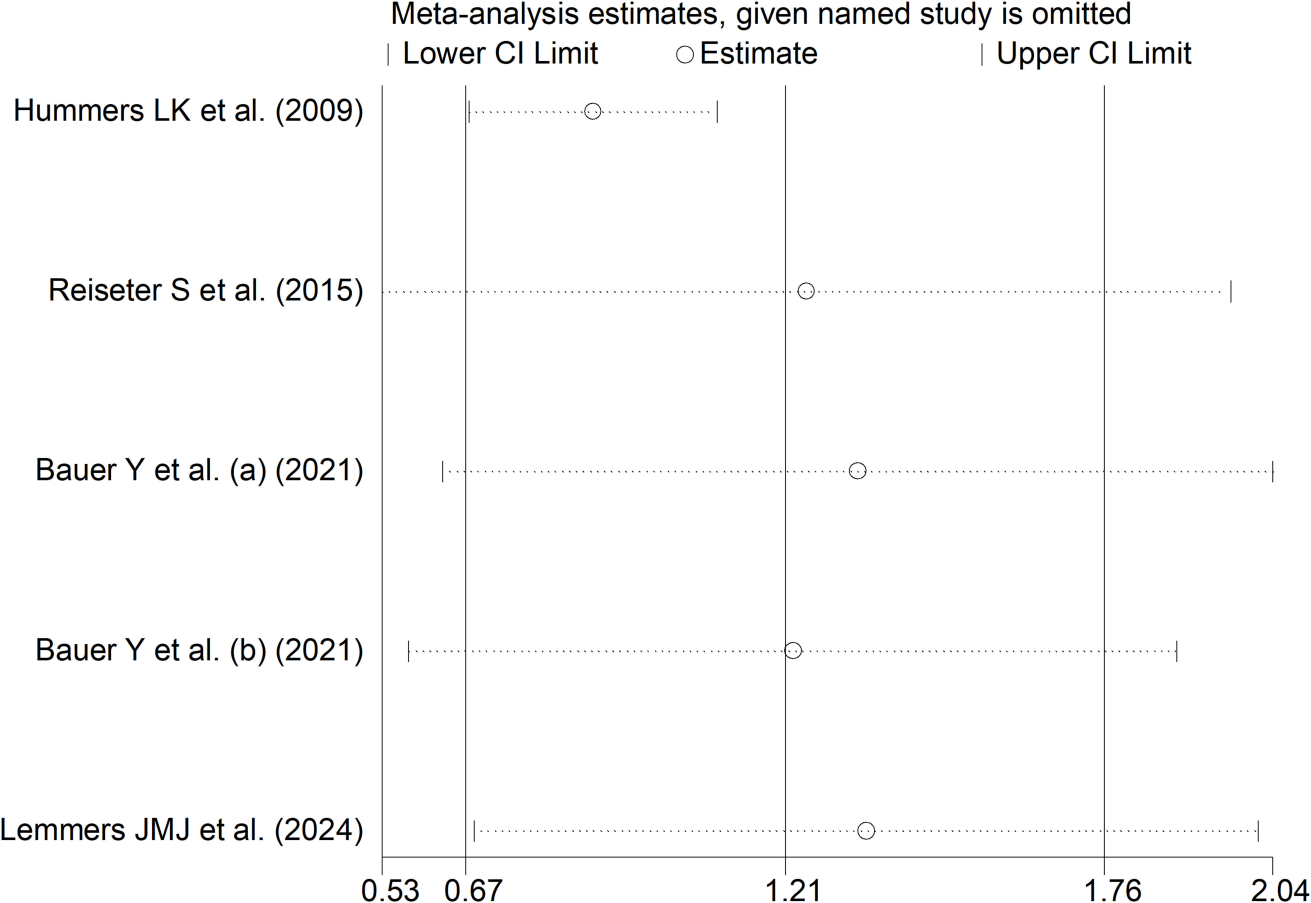
Sensitivity analysis of the association between endostatin concentrations and pulmonary arterial hypertension. For each study, the displayed effect size (hollow circles) and horizontal lines represent the overall effect size and 95% confidence intervals calculated from a meta-analysis excluding that study. The central vertical line represents the overall SMD whereas the lateral vertical lines represent the overall 95% confidence intervals.

The forest plot showed that endostatin concentrations were significantly higher in SSc patients with pulmonary arterial hypertension than those without (SMD=1.21, 95% CI 0.67 to 1.76, p<0.001; I^2^ = 85.6%, p<0.001; **Figure 12**). The results were stable in sensitivity analysis (the effect size ranged between 0.88 and 1.35; **Figure 13**). The overall level of the certainty of evidence was downgraded to very low as the small number of studies prevented the assessment of publication bias, meta-regression and sub-group analyses.

### Interstitial lung disease

One European study with low risk of bias reported endostatin concentrations in 148 SSc patients, 96 without and 52 with interstitial lung disease (42). Non-significant between-group differences were reported (90.1 ± 33 vs. 79.6 ± 20 ng/mL, p=0.16).

### Telangiectasias

One European study with low risk of bias reported endostatin concentrations in 42 SSc patients, 23 without and 19 with telangiectasias (35). Patients with telangiectasias had significantly lower endostatin concentrations than those without (median: 6, IQR 0-750 vs. 20, IQR 4-750 ng/mL, p=0.02).

### Gastrointestinal manifestations

One Asian study with moderate risk of bias investigated endostatin concentrations in 50 SSc patients, 31 without and 19 with gastrointestinal manifestations (49). Non-significant between-group differences were reported (85.3 ± 20.2 vs. 85.4 ± 20.0 ng/mL, p=0.99).

## Discussion

The results of this systematic review and meta-analysis suggest that endostatin is worthy of further investigation in experimental and clinical studies as a candidate biomarker of SSc. We observed significant elevations in the circulating concentrations of this glycoprotein in SSc patients overall and in association with specific complications, i.e., digital ulcers and pulmonary arterial hypertension. By contrast, we observed no significant alterations in endostatin concentrations in other SSc subgroups, specifically in patients with limited vs. diffuse disease and different video capillaroscopy pattern. There was insufficient evidence to evaluate endostatin in SSc patients with other complications, including interstitial lung disease, telangiectasias, and gastrointestinal manifestations. Meta-regression and subgroup analysis of studies investigating endostatin in SSc patients and controls showed no significant associations between the effect size and various patient and study characteristics. Given the generally high between-study heterogeneity and the very low level of the certainty of evidence with most studied endpoints, additional research is warranted to further investigate endostatin and justify its routine use in clinical practice.

The anti-angiogenic effects of endostatin were initially investigated in experimental models of cancer. In seminal studies, this 20-kDa fragment located at the C-terminal of the NC1 domain of the type XVIII collagen α_1_ chain was shown to exert significant and dose-dependent anti-proliferative effects in several endothelial, but not non-endothelial, cell lines. Such effects were associated with reduced tumor growth and metastasis (53). Further research has shown that the anti-angiogenic effects of endostatin are primarily mediated by blocking the binding of VEGF to its receptors, VEGFR-1 and VEGFR-2 (54). Further studies have also reported that endostatin exerts significant anti-fibrotic effects through several mechanisms. Such mechanisms include the downregulation of transforming growth factor β1 and early growth response-1 (20, 55), and the inhibition of the Rhoa/Rho-associated kinase (ROCK) (56), nuclear factor-κB (57), and platelet-derived growth factor pathways (21, 58). Therefore, the combination of anti-angiogenic and anti-fibrotic effects is likely to account for the complex role played by endostatin in SSc, a condition characterized by ineffective angiogenesis and excess fibrosis (1–4).

The biological effects of endostatin on angiogenesis can at least partially explain the significant elevations in endostatin observed in SSc patients with complications characterized by vascular dysfunction and ineffective angiogenesis, e.g., digital ulcers and pulmonary arterial hypertension (59, 60). However, the lack of changes observed in endostatin concentrations according to different video capillaroscopic patterns, a feature associated with disease activity and disease duration (61), suggests that further research is warranted to investigate endostatin in SSc patients with different clinical manifestations, including patients with fibrosis affecting internal organs such as the lung. These issues notwithstanding, the results of a recent study using proteomic analysis support the role of this glycoprotein as a candidate biomarker of SSc. In this study, endostatin concentrations were shown to be significantly associated with disease progression in 55 SSc patients with prospective data up to five years (hazard ratio= 10.2, 95% CI 2.2 to 47.6, p=0.003) (22).

Strengths of our study include the evaluation of endostatin concentrations in a wide range of SSc patient subtypes (e.g., disease type, video capillaroscopy patterns, and specific clinical complications), the assessment of the certainty of evidence, and the evaluation of associations between the effect size and specific study and patient characteristics. Significant limitations are the limited number of studies investigating endostatin in SSc patients with telangiectasia, interstitial lung disease, and gastrointestinal manifestations, as well as the high heterogeneity observed and the very low level of the certainty of evidence with most studied endpoints.

In conclusion, our study has shown significant elevations in endostatin associated with the presence of SSc and specific complications, i.e., digital ulcers and pulmonary arterial hypertension. Further studies are warranted to investigate this candidate biomarker in a wide range of SSc subtypes and the potential influence of other clinically relevant factors, e.g., immunosuppressive treatments, co-morbidities, and disease duration.

## Data Availability

The raw data supporting the conclusions of this article will be made available by the authors, without undue reservation.

## Supplementary material

Supplementary Figure 1 Forest plot of studies investigating endostatin concentrations in patients with systemic sclerosis and healthy controls according to geographical area. The forest plot displays the standardized mean differences (SMDs) and their 95% confidence intervals for each study included in the meta-analysis. Each square represents the effect size of an individual study, with the size of the square proportional to the study’s weight in the analysis. The horizontal lines indicate the 95% confidence intervals for each study, and the vertical line at 0 represents the null effect. The overall pooled effect size is represented by the diamond at the bottom of the plot, with the width of the diamond reflecting the confidence interval. Studies with confidence intervals crossing the null line (0) indicate non-significant results.
