# Supplementary figures and images for: Endostatin as a biomarker of systemic sclerosis: insights from a systematic review and meta-analysis
Source: Front Immunol. 2024 Dec 23;15:1450176. doi: 10.3389/fimmu.2024.1450176 (PMC11701163; doi:10.3389/fimmu.2024.1450176)

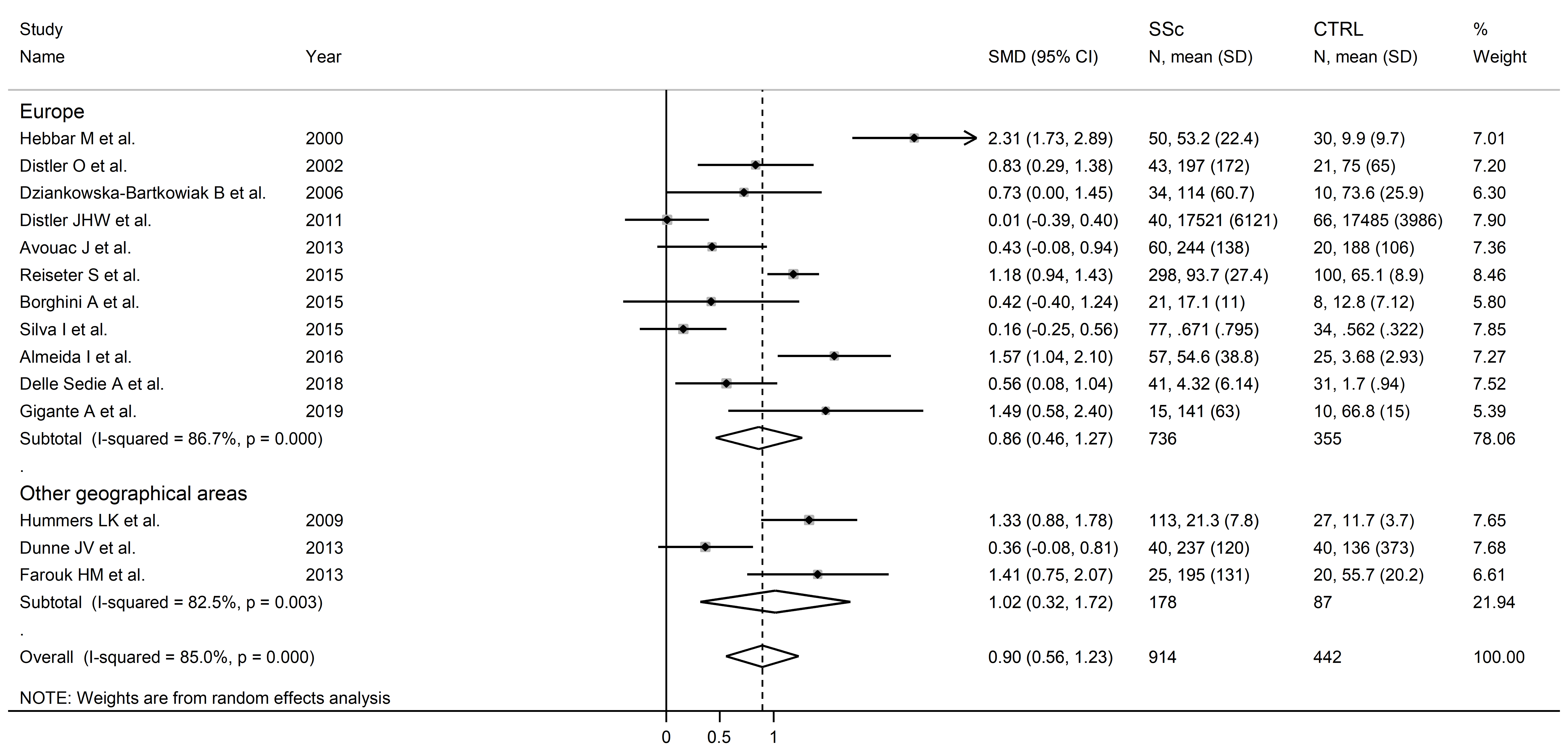

Supplement: Supplementary Figure 1 — Forest plot of studies investigating endostatin concentrations in patients with systemic sclerosis and healthy controls according to geographical area. The forest plot displays the standardized mean differences (SMDs) and their 95% confidence intervals for each study included in the meta-analysis. Each square represents the effect size of an individual study, with the size of the square proportional to the study’s weight in the analysis. The horizontal lines indicate the 95% confidence intervals for each study, and the vertical line at 0 represents the null effect. The overall pooled effect size is represented by the diamond at the bottom of the plot, with the width of the diamond reflecting the confidence interval. Studies with confidence intervals crossing the null line (0) indicate non-significant results. [file Image1.tif]
