# Supplementary material for: Endostatin as a biomarker of systemic sclerosis: insights from a systematic review and meta-analysis
Source: Front Immunol. 2024 Dec 23;15:1450176. doi: 10.3389/fimmu.2024.1450176 (PMC11701163; doi:10.3389/fimmu.2024.1450176)
Supplement: Supplementary file 3 [file Table2.docx]

**Supplementary Table 2.** Assessment of the risk of bias.

| **Study** | **Were the inclusion criteria clearly defined?** | **Were the subjects and the setting described in detail?** | **Was the exposure measured in a reliable way?** | **Were standard criteria used to assess the condition?** | **Were confounding factors identified?** | **Were strategies to deal with confounding factors stated?** | **Were the outcomes measured in a reliable way?** | **Was appropriate statistical analysis used?** | **Risk of bias** |
| --- | --- | --- | --- | --- | --- | --- | --- | --- | --- |
| Hebbar M et al. (1) | No | Yes | Yes | Yes | No | No | Yes | Yes | Moderate |
| Distler O et al. (2) | Yes | Yes | Yes | Yes | No | No | Yes | Yes | Low |
| Dziankowska-Bartkowiak B et al. (3) | No | No | Yes | Yes | No | No | Yes | Yes | Moderate |
| Hummers LK et al. (4) | No | Yes | Yes | Yes | Yes | Yes | Yes | Yes | Low |
| Distler JH et al. (5) | No | Yes | Yes | Yes | Yes | Yes | Yes | Yes | Low |
| Avouac J et al. (6) | No | Yes | Yes | Yes | Yes | Yes | Yes | Yes | Low |
| Dunne JV et al. (7) | Yes | Yes | Yes | Yes | No | No | Yes | Yes | Low |
| Farouk HM et al. (8) | No | Yes | Yes | Yes | No | No | Yes | Yes | Moderate |
| Reiseter S et al. (9) | No | Yes | Yes | Yes | Yes | Yes | Yes | Yes | Low |
| Ribatti D et al. (10) | No | Yes | Yes | Yes | Yes | No | No | Yes | Moderate |
| Silva I et al. (11) | Yes | Yes | Yes | Yes | Yes | Yes | Yes | Yes | Low |
| Almeida I et al. (12) | No | Yes | Yes | Yes | Yes | No | No | Yes | Moderate |
| Gigante A et al. (13) | Yes | Yes | Yes | Yes | No | No | Yes | Yes | Low |
| Delle Sedie A et al. (14) | No | Yes | Yes | Yes | No | No | Yes | Yes | Moderate |
| Gigante A et al. (15) | Yes | Yes | Yes | Yes | No | No | Yes | Yes | Low |
| Mecoli CA et al. (16) | Yes | Yes | Yes | Yes | No | No | Yes | Yes | Low |
| Nakane S et al. (17) | No | Yes | Yes | Yes | No | No | Yes | Yes | Moderate |
| Bauer T et al. (18) | No | Yes | Yes | Yes | No | No | Yes | Yes | Moderate |
| Lemmers JM et al. (19) | Yes | Yes | Yes | Yes | No | No | Yes | Yes | Low |

**References**

1. Hebbar M, Peyrat JP, Hornez L, Hatron PY, Hachulla E, Devulder B. Increased concentrations of the circulating angiogenesis inhibitor endostatin in patients with systemic sclerosis. Arthritis Rheum. 2000;43(4):889-93. doi: 10.1002/1529-0131(200004)43:4<889::AID-ANR21>3.0.CO;2-5

2. Distler O, Del Rosso A, Giacomelli R, Cipriani P, Conforti ML, Guiducci S, et al. Angiogenic and angiostatic factors in systemic sclerosis: increased levels of vascular endothelial growth factor are a feature of the earliest disease stages and are associated with the absence of fingertip ulcers. Arthritis Res. 2002;4(6):R11. doi: 10.1186/ar596

3. Dziankowska-Bartkowiak B, Zalewska A, Sysa-Jedrzejowska A. Duration of Raynaud's phenomenon is negatively correlated with serum levels of interleukin 10 (IL-10), soluble receptor of interleukin 2 (sIL2R), and sFas in systemic sclerosis patients. Med Sci Monit. 2004;10(5):CR202-8. doi:

4. Hummers LK, Hall A, Wigley FM, Simons M. Abnormalities in the regulators of angiogenesis in patients with scleroderma. J Rheumatol. 2009;36(3):576-82. doi: 10.3899/jrheum.080516

5. Distler JH, Strapatsas T, Huscher D, Dees C, Akhmetshina A, Kiener HP, et al. Dysbalance of angiogenic and angiostatic mediators in patients with mixed connective tissue disease. Ann Rheum Dis. 2011;70(7):1197-202. doi: 10.1136/ard.2010.140657

6. Avouac J, Vallucci M, Smith V, Senet P, Ruiz B, Sulli A, et al. Correlations between angiogenic factors and capillaroscopic patterns in systemic sclerosis. Arthritis Research & Therapy. 2013;15(2). doi: 10.1186/ar4217

7. Dunne JV, Keen KJ, Van Eeden SF. Circulating angiopoietin and Tie-2 levels in systemic sclerosis. Rheumatol Int. 2013;33(2):475-84. doi: 10.1007/s00296-012-2378-4

8. Farouk HM, Hamza SH, El Bakry SA, Youssef SS, Aly IM, Moustafa AA, et al. Dysregulation of angiogenic homeostasis in systemic sclerosis. Int J Rheum Dis. 2013;16(4):448-54. doi: 10.1111/1756-185X.12130

9. Reiseter S, Molberg Ø, Gunnarsson R, Lund MB, Aalokken TM, Aukrust P, et al. Associations between circulating endostatin levels and vascular organ damage in systemic sclerosis and mixed connective tissue disease: an observational study. Arthritis Research & Therapy. 2015;17(1). doi: 10.1186/s13075-015-0756-5

10. Ribatti D, Borghini A, Manetti M, Nacci F, Bellando-Randone S, Guiducci S, et al. Systemic Sclerosis Sera Impair Angiogenic Performance of Dermal Microvascular Endothelial Cells: Therapeutic Implications of Cyclophosphamide. Plos One. 2015;10(6). doi: 10.1371/journal.pone.0130166

11. Silva I, Teixeira A, Oliveira J, Almeida I, Almeida R, Vasconcelos C. Predictive value of vascular disease biomarkers for digital ulcers in systemic sclerosis patients. Clin Exp Rheumatol. 2015;33(4 Suppl 91):S127-30. doi:

12. Almeida I, Oliveira Gomes A, Lima M, Silva I, Vasconcelos C. Different contributions of angiostatin and endostatin in angiogenesis impairment in systemic sclerosis: a cohort study. Clin Exp Rheumatol. 2016;34 Suppl 100(5):37-42. doi:

13. Gigante A, Margiotta D, Navarini L, Barbano B, Gasperini ML, D'Agostino C, et al. Serum level of endostatin and digital ulcers in systemic sclerosis patients. Int Wound J. 2018;15(3):424-8. doi: 10.1111/iwj.12882

14. Delle Sedie A, Riente L, Maggiorini L, Pratesi F, Tavoni A, Migliorini P, et al. Potential biomarkers in patients with systemic sclerosis. International Journal of Rheumatic Diseases. 2017;21(1):261-5. doi: 10.1111/1756-185x.13196

15. Gigante A, Navarini L, Margiotta D, Barbano B, Afeltra A, Rosato E. Female sexual dysfunction in systemic sclerosis: The role of endothelial growth factor and endostatin. J Scleroderma Relat Disord. 2019;4(1):71-6. doi: 10.1177/2397198318776593

16. Mecoli CA, Perin J, Van Eyk JE, Zhu J, Fu Q, Allmon AG, et al. Vascular biomarkers and digital ulcerations in systemic sclerosis: results from a randomized controlled trial of oral treprostinil (DISTOL-1). Clin Rheumatol. 2020;39(4):1199-205. doi: 10.1007/s10067-019-04863-0

17. Nakane S, Umeda M, Kawashiri SY, Mukaino A, Ichinose K, Higuchi O, et al. Detecting gastrointestinal manifestations in patients with systemic sclerosis using anti-gAChR antibodies. Arthritis Res Ther. 2020;22(1):32. doi: 10.1186/s13075-020-2128-z

18. Bauer Y, de Bernard S, Hickey P, Ballard K, Cruz J, Cornelisse P, et al. Identifying early pulmonary arterial hypertension biomarkers in systemic sclerosis: machine learning on proteomics from the DETECT cohort. Eur Respir J. 2021;57(6). doi: 10.1183/13993003.02591-2020

19. Lemmers JM, van Caam AP, Kersten B, van den Ende CH, Knaapen H, van Dijk AP, et al. Nailfold capillaroscopy and candidate-biomarker levels in systemic sclerosis-associated pulmonary hypertension: A cross-sectional study. J Scleroderma Relat Disord. 2023;8(3):221-30. doi: 10.1177/23971983231175213
